# Attenuation of diet-induced hypothalamic inflammation following bariatric surgery in female mice

**DOI:** 10.1186/s10020-018-0057-y

**Published:** 2018-10-24

**Authors:** Mary K. Herrick, Kristin M. Favela, Richard B. Simerly, Naji N. Abumrad, Nathan C. Bingham

**Affiliations:** 1 Department of Pediatrics, Vanderbilt University Medical Center, 1500 21 st. Ave South, Suite 1514, Nashville, TN 37212 USA; 2 Present address: Department of Physiology, Emory University School of Medicine, Atlanta, GA 30322 USA; 3 Department of Molecular Physiology and Biophysics, Vanderbilt University Medical Center, Nashville, TN 37232 USA; 4 Department of Surgery, Vanderbilt University Medical Center, Nashville, TN 37232 USA

**Keywords:** Obesity, Bariatric surgery, Hypothalamus, Inflammation, Microglia

## Abstract

**Background:**

Exposure of rodents to chronic high-fat diet (HFD) results in upregulation of inflammatory markers and proliferation of microglia within the mediobasal hypothalamus. Such hypothalamic inflammation is associated with metabolic dysfunction, central leptin resistance, and maintenance of obesity. Bariatric surgeries result in long-term stable weight loss and improved metabolic function. However, the effects of such surgical procedures on HFD-induced hypothalamic inflammation are unknown. We sought to characterize the effects of two bariatric surgical procedures, Roux-en-Y gastric bypass (RYGB) and biliary diversion (BD-IL), in female mice with particular emphasis on HFD-induced hypothalamic inflammation and microgliosis.

**Methods:**

RYGB and BD-IL were performed on diet-induced obese (DIO) mice. Quantitative RT-PCR and fluorescent microscopy were used to evaluate hypothalamic inflammatory gene expression and microgliosis. Results were compared to lean (CD), DIO sham-surgerized mice (DIO-SHAM), and dietary weight loss (DIO-Rev) controls.

**Results:**

In female mice, RYGB and BD-IL result in normalization of hypothalamic inflammatory gene expression and microgliosis within 8 weeks of surgery, despite ongoing exposure to HFD. Paralleling these results, the hypothalamic expression levels of the orexigenic neuropeptide *Agrp* and the anorexic response of surgical mice to exogenous leptin were comparable to lean controls (CD). In contrast, results from DIO-Rev mice were comparable to DIO-SHAM mice, despite transition back to standard rodent show and normalization of weight.

**Conclusion:**

Bariatric surgery attenuates HFD-induced hypothalamic inflammation and microgliosis and restores leptin sensitivity, despite ongoing exposure to HFD.

## Background

It is well established, in human and rodent models, that caloric excess and the ensuing diet-induced obesity (DIO), result in a state of chronic, low-grade inflammation and accumulation of professional immune cells such as macrophages in metabolic tissues including liver, adipose and muscle. This inflammatory state is often termed ‘metaflammation’ (metabolically induced inflammation) to distinguish it from the more acute and high-grade inflammation associated with injury and infection that more often results in appetite suppression and weight loss. In peripheral tissues, metaflammation has been linked to metabolic dysfunction including insulin resistance (Hotamisligil et al. 1993; Kern et al. 1995). More recent work has shown a similar condition occurring in the mediobasal hypothalamus (MBH), an important center of neuronal control of energy homeostasis. With exposure to high-fat diet (HFD), hypothalamic inflammatory pathways, such as NF-κB, are activated and the expression of pro-inflammatory mediators, including canonical proinflammatory cytokines, *Il-1β* and *Tnf*α are upregulated (De Souza et al. 2005; Zhang et al. 2008). Comparable to that observed in peripheral tissues, hypothalamic metaflammation is accompanied by accumulation of microglia, the resident immune cells of the central nervous system (Thaler et al. 2012a). Such reactive microgliosis is generally a response to central nervous system injury and is associated with a transition of microglia from a resting or surveillance state to a more active state accompanied by the production of immune response molecules (Streit et al. 1999). Importantly, hypothalamic metaflammation and microglial activation contribute to hypothalamic resistance to peripheral anorexic hormones such as leptin, thus increasing the threshold for leptin’s catabolic effects and contributing to an elevated level of homeostatically defended body weight (Zhang et al. 2008). Both pharmacologic and genetic experimental interventions that inhibit hypothalamic inflammatory pathways reduce food intake and body weight and improve the response of obese animals to exogenous leptin (Zhang et al. 2008). These findings suggest that therapies designed to abrogate hypothalamic metaflammation may prove valuable in the treatment of obesity.

Currently, bariatric surgery has emerged as the most effective obesity treatment available in both magnitude and durability of its effects (Mingrone et al. 2012; Schauer et al. 2017). Roux-en-Y gastric bypass (RYGB), one of the most effective and commonly performed procedures, involves creation of a smaller stomach pouch while diverting nutrient flow to varying distal segments of the intestine. Studies from the past 10–15 years have shown that RYGB, while initially designed to produce weight loss through a combination of gastric restriction and malabsorption, clearly has metabolic benefits independent from these intended mechanisms of action (Albaugh et al. 2016). Importantly, there is mounting evidence that bariatric surgeries result in a downward shift in the level of homeostatically defended body weight. In humans, RYGB leads to a decrease in hunger and preference for calorically-dense foods despite the significant decrease in serum leptin levels, an environment that should generally induce hyperphagia (Laurenius et al. 2013; Ullrich et al. 2013; Beckman et al. 2010). Following bariatric surgery, rodents that are induced to gain weight via pharmacologic blockade of central melanocortin receptors, rapidly return to their stable, post-operative body weight after removal of the blockade. Similar results are seen in female mice following pregnancy (Grayson et al. 2013; Munzberg et al. 2015). These studies demonstrate that despite the physical ability to increase food intake following bariatric surgery, rodents choose to eat less and defend a lower body weight. Given the known effects of hypothalamic metaflammation on hypothalamic leptin resistance, these studies raise the possibility that bariatric surgery may improve hypothalamic metaflammation contributing to a lower set point of defended body weight.

In this study, we use two mouse models to investigate the effects of bariatric surgery on hypothalamic metaflammation. In addition, given that the large majority of bariatric surgery patients are female (Pratt et al. 2009) we evaluated these effects in female mice, an underutilized model. We demonstrate that HFD-induced hypothalamic metaflammation and microgliosis persist in DIO mice, even after reverting back to a low-fat diet and loss of excess weight, while bariatric surgery results in a rapid normalization of both parameters.

## Methods

### Animals and diets

**Fig. 1:**
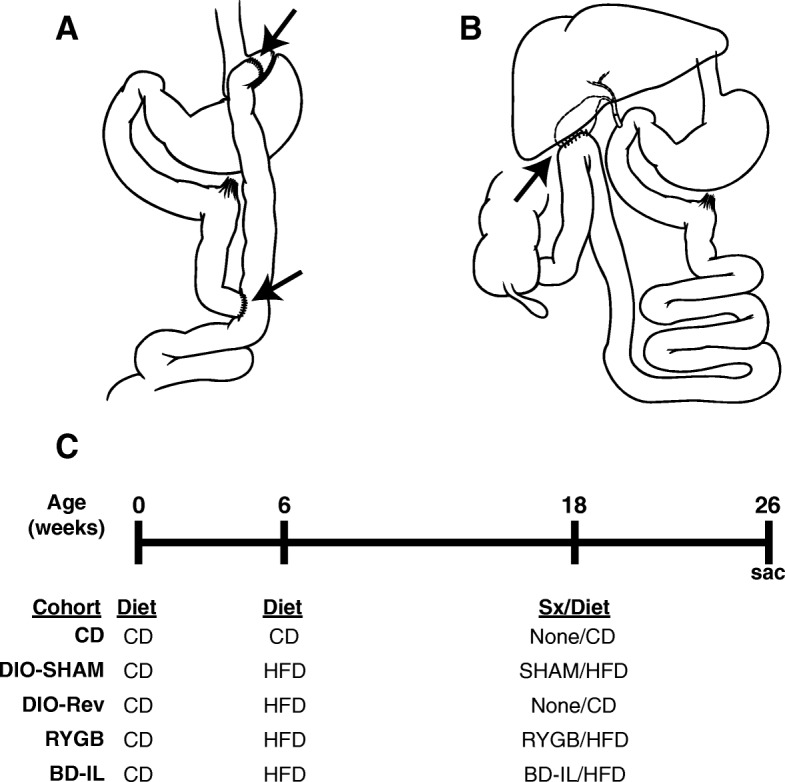
Models of murine bariatric surgery and experimental design. **a** For Roux-en-Y gastric bypass (RYBG) procedure, the stomach was ligated between the gastric fundus (forestomach) and glandular portion while the jejunum was transected 4 cm from the Ligament of Treitz. The distal jejunum was subsequently anastomosed to the forestomach (top arrow) with GI continuity being maintained via a jejuno-jejunostomy 6 cm distal from the initial transection (lower arrow). Image modified from (Albaugh et al. 2016). **b** For the biliary diversion procedure (BD-IL), the common bile duct was ligated proximal to the pancreatic duct and the gallbladder anastomosed to the ileum 4 cm proximal to the ileo-cecal valve. Image modified from (Albaugh et al. 2016). **c** Experimental design. Age of mice shown in weeks with time of dietary changes and surgical procedures (Sx) shown at 6 and 18 weeks. All mice were sacrificed at 26 weeks of age

Female C57BL/6J and CX3CR1^GFP^ mice, on a C57BL/6J background (> 12 generations) were obtained from Jackson Laboratory (Stock no. 000664 and 005582, respectively). A cohort of CX3CR1^GFP^ mice were used for microglial quantification, while all other experiments were performed using wild-type C57BL/6J mice. All experimental mice were bred in-house and housed at 23 °C on a 12-h light cycle. From birth until 6 weeks of age, mice were given free access to a standard rodent chow (PicoLab® Laboratory Rodent Diet 5L0D, 13.4% kcal from fat; Land O’Lakes Inc., St. Louis, MO, USA). Beginning at 6 weeks of age, mice were randomly allocated to each of five experimental groups 1) control diet (CD), 2) diet-induced obese sham (DIO-SHAM), 3) diet-induced obese reversal (DIO-Rev), 4) Roux-en-Y gastric bypass (RYGB), or 5) biliary diversion to the ileum (BD-IL). At that time, all groups, except for CD controls, were transitioned to a HFD (60% kcal from fat, Research Diets, D12492, New Brunswick, NJ) for 12 weeks. At 18 weeks of age, surgical groups underwent their respective surgical procedures and remained on HFD. DIO-Rev animals were transitioned back to the standard rodent chow while CD animals remained on standard rodent chow (Fig. 1c). All mice were weighed weekly and fed ad libitum for an additional 8 weeks before being sacrificed for analysis.

### Bariatric surgery

RYGB, BD-IL, and sham surgical procedures were performed at 18 weeks of age under isoflurane anesthesia using a 12-15X microsurgical scope as previously described (Flynn et al. 2015; Yin et al. 2011). Briefly, for RYGB, the stomach was ligated between the gastric fundus (forestomach) and glandular portion while the jejunum was transected 4 cm from the Ligament of Treitz. The distal jejunum was subsequently anastomosed to the forestomach with GI continuity maintained via a jejuno-jejunostomy 6 cm distal from the initial transection (Fig. 1a). BD-IL was performed by ligating the common bile duct and creating a gallbladder to ileum anastomosis 4 cm proximal to the ileo-cecal valve (Fig. 1b). The sham surgical procedure was performed in parallel to RYGB with similar abdominal incision, physical manipulation of stomach and intestine (without transection or re-anastomosis), and suturing.

### Body weight and composition

Body mass was measured using mq10 NMR analyzer (Bruker Optics Inc., Billerica, MA) following 2 h of fasting. Fat and muscle mass were calculated in grams.

### Glucose tolerance tests

After a 6 h fast, mice were given a 1.5 mg/g dose of glucose by oral gavage. Blood glucose was measured using a hand-held glucometer (Accu-chek, Roche Diagnostic Corporation, Indianapolis, IN) at 0, 15, 30, 60 and 120 min.

### Intraperitoneal leptin treatment

Mice were individually housed and sham injected for 5 days with intraperitoneal (i.p.) saline prior to drug treatment. Subsequently, mice received i.p. injections of recombinant murine leptin (Peprotech, 2.0 μg/g body weight) for 3 days. Food intake was measured daily. Caloric intake during the 72 h after first leptin injection was compared to the last 72 h of saline injections.

### Microglial quantification

Female heterozygous CX3CR1^GFP^ mice were allocated to surgical and control groups as described above. Deeply anesthetized mice were transcardially perfused with ~ 10–15 ml of 0.1 M PBS (pH 7.4) followed by 50 ml of ice-cold 4% paraformaldehyde (PFA) in 0.1 M PBS at 4 °C. Brains were carefully dissected and postfixed overnight in 4% PFA at 4 °C then equilibrated for 24-48 h with 30% sucrose in 0.1 M PBS. Twenty-five micron sections were obtained using a sliding microtome (Leica Microsystems, Deerfield, IL) with frozen stage set at − 18 °C (Physitemp, Clifton, NJ). Sections were stored free-floating at − 20 °C in cryoprotectant (30% ethylene glycol, 30% glycerol, in 0.1 M PBS). Subsequently, sections were washed in PBS and counterstained with DAPI before being mounted onto Superfrost Plus microscope slides (Fisher Scientific, Walham, MA) and coverslipped using Prolong Gold antifade mounting media (Invitrogen, La Jolla, CA).

Four to five mice were used from each experimental cohort with two to three sections from the mediobasal hypothalamus used for analysis (Bregma − 1.6 to − 1.9. Slides were scanned using a Leica Aperio Scanscope FL with 20X magnification. Bilateral arcuate (ARC), ventromedial (VMH), and dorsomedial (DMH) nuclei were manually outlined and annotated using Aperio ImageScope software. Bilateral areas of the retrosplenial cortex, just lateral to the longitudinal fissure, were used as nonhypothalamic control areas. GFP+ microglia in each area were quantified using the CytoNuclear algorithm (v1.4, Indica Laboratories) within the Aperio eSlide Manager platform and the number of positive cells was normalized to the area of each annotated region. Representative images of HFD-induced ARC microgliosis were obtained using a laser-scanning confocal microscope (Leica TCS SPE confocal microscope) equipped with a 40x oil-corrected objective. Image stacks (20 μm thick) were collected through the z-axis at a frequency of 0.5 μm.

### Quantitative real-time PCR

Female C57BL/6J mice were allocated to surgical and control groups as described above. Deeply anesthetized mice were transcardially perfused with ~ 10–15 ml of 0.1 M PBS (pH 7.4) at 4 °C and brains were carefully dissected. Using a brain matrix (Braintree Scientific, Braintree, MA), a 1 mm coronal section was obtained and the mediobasal hypothalamus removed under dissecting microscope. Hypothalami where placed directly in tissue lysis solution and snap frozen on dry ice. Samples were kept at − 80 °C until RNA extraction using a commercially available kit according to manufacturer specifications (RNAqueous-Micro Kit; Ambion). RNA was amplified and reverse transcribed using the Ovation RNA amplification kit (Nugen). Semiquantitive PCR was performed on a QuantStudio 3 Real-Time PCR System (Applied Biosystems, Foster City, CA) using gene-specific Taqman probes (Applied Biosystems). The transcripts assayed with their NCBI reference sequence and Taqman assay IDs (RefSeq, ID) are as follows: *Gapdh*, (NM_001289726.1, Mm99999915_g1); *Rn18s* (NR_003278.3 m, Mm03928990_g1); *Tnf* (NM_013693.3, Mm00443258_m1); *Il1b* (NM_008361.3, Mm00434228_m1); *Ccl2* (NM_011333.3, Mm00441242_m1); *Pomc* (NM_001278584.1, Mm00435874_m1); Agrp (NM_007427.3, Mm00475829_g1). Expression levels of each gene were normalized to reference genes (*Gapdh* and *18S*) and expressed relative to DIO-controls using the ∆∆CT method.

### Statistics

Statistical analysis was performed using Prism Statistical Software (v6.07, GraphPad Software, La Jolla, CA). Means were analyzed by either unpaired *t* test or analysis of variance (ANOVA) and appropriate post hoc analyses. All data are expressed as mean ± SEM with *P* < 0.05 considered significant.

## Results

### Biliary diversion to the ileum (BD-IL), roux-en-Y gastric bypass (RYGB), and diet-induced obesity reversal (DIO-rev) result equivalent weight loss and improved glucose tolerance

**Fig. 2:**
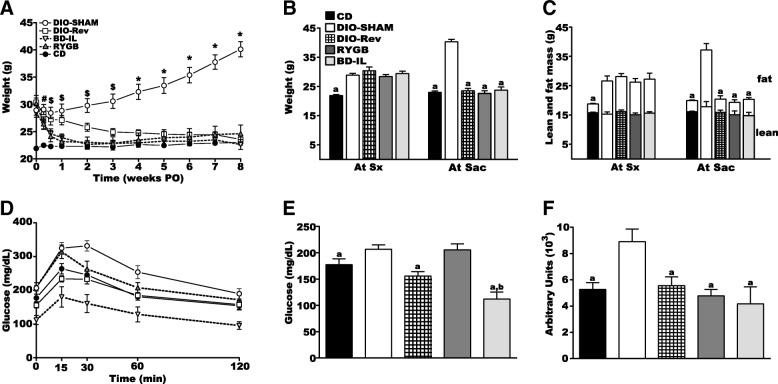
Bariatric surgery result in normalization of body weight, body composition, and glucose tolerance. **a** Body weight over time following bariatric surgery or diet-reversal. At the time of surgery (At sx) mice are 18 weeks of age with the weight of all groups statistically different from CD. The weights of both RYGB and BD-IL mice reached similar weights to CD controls approximately 4 days after surgery while DIO-Rev mice did so 4 weeks after diet transition. # *p* < 0.05 for DIO-SHAM, DIO-Rev, and RYGB versus CD. $ p < 0.05 for DIO-SHAM and DIO-Rev versus CD. * *p* < 0.05 for DIO-SHAM versus CD. **b** Body weight of all experimental groups at the time of surgery (At Sx) and sacrifice (At Sac). **c** Body composition of all experimental groups at the time of surgery (At Sx) and sacrifice (At Sac). **d** Blood glucose levels in surgical and control mice during an oral glucose tolerance test. **e** Fasting blood glucose levels (**f**) Glucose integrated area under the curve (AUC). a = significant difference versus DIO-SHAM. b = significant difference versus CD. *p* < 0.05 (*n* = 6–8 per group). Data are represented as mean ± SEM

Both BD-IL and RYGB have been shown to effectively induce and maintain weight loss in male DIO mice (Flynn et al. 2015). To evaluate the ability of these procedures to ameliorate metabolic parameters in female mice, we compared surgical mice to DIO sham-surgerized (DIO-SHAM) and lean controls (CD), as well as DIO mice transitioned from high-fat diet to standard chow diet (DIO-Rev) (Fig. 1a). All mice, except for CD mice, were transitioned to HFD at 6 weeks of age. After 12 weeks of HFD feeding, HFD mice weighed approximately 8 g more than the CD controls (Fig. 2b), with the excess weight entirely accounted for by increased adipose mass (Fig. 2c). DIO mice were then randomly assigned to a surgical (BD-IL, RYGB, or DIO-SHAM) or the diet reversal (DIO-Rev) cohort. All surgical mice were continued on their pre-surgical high-fat diet while DIO-Rev mice were transitioned back to standard rodent chow fed ad libitum (Fig. 1c).

Following surgery, BD-IL and RYGB mice rapidly normalized their body weight and were statistically similar to CD controls within one week (Fig. 2a). Both surgical groups maintained a stable, lower body weight, equivalent to CD controls, through the end of the study (Fig. 2a,b) without any significant (< 5%) mortality outside of the immediate post-operative period (10 days). DIO-Rev animals also normalized their body weight, albeit at a slower rate than their surgical counterparts, reaching statistical equivalency to the CD controls after ~ 4 weeks post diet reversal (Fig. 2a). To assure a stable, steady-state weight, mice were maintained for an additional 4 weeks before further testing or analysis. At the end of the study, DIO-SHAM mice were approximately 16 g heavier than their surgical or control counterparts. Body composition analysis showed that neither surgical procedure resulted in a loss of lean body mass and differences in weight between groups was again almost entirely accounted for by adipose mass, with no statistical difference in lean body mass seen between cohorts (Fig. 2c).

Eight weeks following surgery all cohorts were subjected to oral glucose tolerance tests (Fig. 2d-f). DIO-SHAM animals demonstrated significant glucose intolerance with both elevated fasting and glucose area under the curve. DIO-Rev animals normalized their glucose tolerance while BD-IL surgical animals showed a significant decrease in both fasting and stimulated glucose levels well below that of CD controls. Interestingly, after a six hour fast, RYGB animals had glucose levels similar to DIO-SHAM animals, although their glucose dynamics where significantly improved. Following, administration of glucose, RYGB animals demonstrated a brisk rise in glucose levels with a peak similar to that of DIO-SHAM. However, this was followed by rapid return to baseline levels, such that the glucose area under the curve was similar to CD fed animals.

### BD-IL and RYGB bariatric surgeries normalize HFD-induced hypothalamic microgliosis

**Fig. 3:**
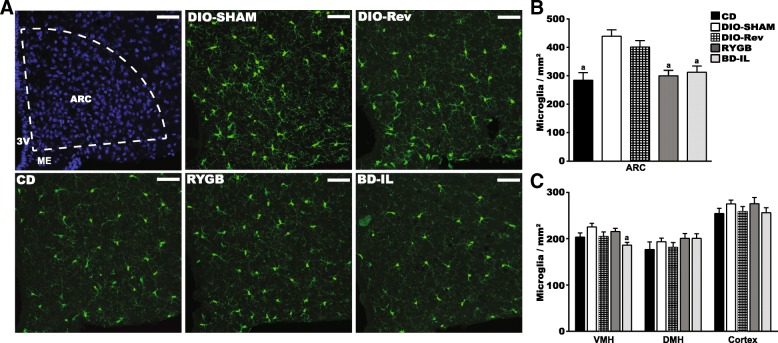
RYGB and BD-IL procedures normalize HFD-induced hypothalamic microgliosis. **a** Representative images of GFP fluorescence from bariatric surgical mice and controls taken at Bregma − 1.6 to − 1.9. Top left image shows DAPI staining of mediolbasal hypothalamus at this level, with the dashed lines defining the anatomical borders of the arcuate nucleus (ARC). 3 V, third ventricle. ME, median eminence. Scale bar = 50 μm. **b** Quantification of microglia in the arcuate nucleus following sacrifice (*n* = 4–5 mice per group, 2–3 sections per mouse). **c** Quantification of microglia in other mediobasal hypothalamic nuclei as well as cortex. a = significant difference from DIO-SHAM. *p* < 0.05 (*n* = 5–6 per group). Data are represented as mean ± SEM

Chronic exposure to HFD results in a reactive microgliosis and increased inflammatory gene expression within the mediobasal hypothalamus of (Thaler et al. 2012a), a condition associated with central leptin resistance (Flynn et al. 2015). Given that BD-IL and RYGB animals maintain a stable normal level of body weight and adiposity without hyperphagia, despite continued exposure to HFD, we hypothesized that these surgeries might improve or resolve hypothalamic metaflammation. Following the same experimental structure (12-week HDF feeding, surgery, and sacrifice at 8 weeks post-operative), we first evaluated the effects of RYGB and BD-IL surgeries on HFD-induced microgliosis. To facilitate identification and visualization of hypothalamic microglia, we used CX3CR1^GFP/+^ mice, a model that uses the CX3CR1 promoter to drive GFP expression in microglia. Using fluorescent microscopy, we found that, consistent with previous reports, chronic HFD-feeding resulted in a significant increase in arcuate microglia numbers (Thaler et al. 2012a; Valdearcos et al. 2014). Both RYGB and BD-IL surgical procedures resulted in a complete normalization of arcuate nucleus microglial cell counts, comparable to CD controls (Fig. 3a,b). Surprisingly, while we observed a trend towards decreased arcuate microglia numbers following diet reversal, this was not statistically different from that of DIO-SHAM animals. In comparison to the ARC, we did not observe any effects of HFD-feeding on microglial counts within nearby hypothalamic nuclei (VMH, DMH) or in the cortex (Fig. 3c). Using multi-comparisons analysis, we did note a decrease of microglia within the VMH of BD-IL animals, which was statistically lower than DIO-SHAM animals.

### BD-IL and RYGB bariatric surgeries normalize HFD-induced hypothalamic metaflammation

**Fig. 4:**
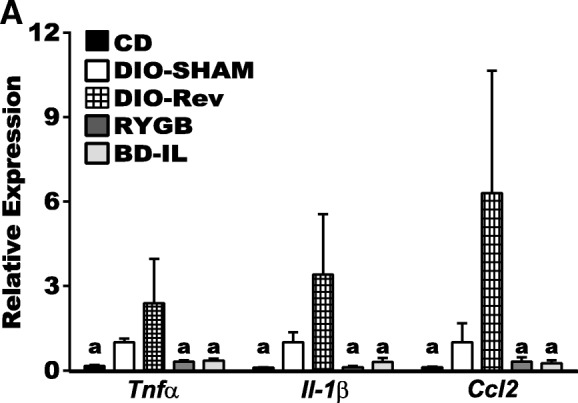
RYGB and BD-IL procedures normalize hypothalamic inflammatory gene expression. **a** Hypothalamic expression by qRT-PCR of pro-inflammatory genes relative to DIO-SHAM control. a = significant difference from DIO-SHAM. *p* < 0.05 (*n* = 5–8 per group). Data are represented as mean ± SEM

Given the improvement in microgliosis, we next evaluated hypothalamic proinflammatory gene expression in wild-type C57BL/6J mice following bariatric surgery. At baseline, we found the expression levels of the proinflammatory genes assayed, including *Tnfα*, *Il-1β*, and *Ccl2*¸ to be extremely low in hypothalamus of the CD control animals, with most samples failing to reach a cycle threshold (Ct) using specific Taqman probes to the genes of interest. In contrast, all assays run on DIO-SHAM samples readily amplified and detected all three genes assayed. Consistent with the pattern seen in microgliosis, hypothalamic proinflammatory gene expression from bariatric surgical samples resembled that of CD controls, with very low detection. We found that weight loss resulting from diet reversal (DIO-Rev) alone did not significantly reduce, but surprisingly led to a general increase in the levels of hypothalamic proinflammatory gene expression, although we saw broad variation. In order to more readily compare hypothalamic gene expression levels between experimental groups, expression levels of each gene were normalized to a reference gene (*Gapdh*) and expressed relative to DIO-controls using the ∆∆CT method, with undetectable samples assigned a Ct value of 35, the latest threshold value seen across all samples assayed. These results suggest that both RYGB and BD-IL procedures promote an anti-inflammatory profile within the hypothalamus of HFD-fed mice while weight loss alone, at least in the time frame assayed here, does not result in resolution of hypothalamic inflammation (Fig. 4a).

### BD-IL and RYGB bariatric surgeries normalize expression of hypothalamic orexigenic gene expression and restores the anorexic response to exogenous leptin

**Fig. 5:**
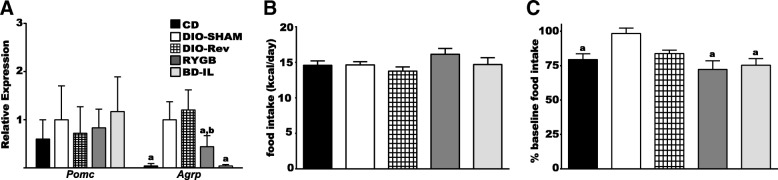
Hypothalamic neuropeptide gene expression and anorexic response to RYGB and BD-IL bariatric surgery. **a** Expression levels of hypothalamic neuropeptides (**b**) Baseline food intake measured over three days and expressed in kcal/day per mouse. **c** Anorexic response to exogenous leptin. a = significant difference from DIO-SHAM. b = significant difference from CD. *p* < 0.05 (*n* = 6–8 per group). Data are represented as mean ± SEM

Because hypothalamic metaflammation is associated with leptin resistance within the MBH (Zhang et al. 2008; Milanski et al. 2009; Posey et al. 2009), we hypothesized that the improved hypothalamic inflammatory profile might be associated with changes in the expression of leptin-responsive genes and an improved response to exogenous leptin. We found that expression of the orexigenic neuropeptide *Agrp* was significantly upregulated in the DIO-SHAM animals compared to CD controls (Fig. 5a). Similar to previous reports, we found that weight loss associated with diet reversal was also associated with increased expression of *Agrp* (Yu et al. 2009). However, following both RYGB and BD-IL, expression levels of hypothalamic *Agrp* were normalized to similar levels found in CD controls. Evaluation of *Pomc* levels, the pro-gene of the anorexigenic neuropeptide α-MSH, showed no significant difference between any of the experimental groups (Fig. 5a). Taken together these results indicate a shift towards a more anorexigenic profile in the hypothalamus and suggest an improved sensitivity to endogenous leptin in HFD-fed mice following bariatric surgery.

Finally, we assayed the response of bariatric surgical animals to exogenous leptin. All animal cohorts where given 3 days of vehicle injections followed by three days of leptin (2 mg/kg) and food intake measured daily. Consistent with previous reports, DIO-SHAM animals proved to be generally resistant to the anorexic effects of leptin, with little reduction of food intake in response to treatment (Fig. 5b). Both the surgical and CD cohorts, however, reduced their intake by 20–25%, while the DIO-Rev again showed an intermediate phenotype. In response to leptin treatment, DIO-Rev animals showed a clear trend towards reduction of food intake (~ 15%), however analysis of variance with multiple comparisons did not show a significant difference between DIO-SHAM and DIO-Rev animals, nor a difference between CD and DIO-Rev animals.

## Discussion

The induction of hypothalamic metaflammation and microgliosis in rodents following chronic HFD-feeding, has been extensively documented and confirmed by our studies here (De Souza et al. 2005; Zhang et al. 2008; Thaler et al. 2012a; Milanski et al. 2009; Posey et al. 2009). Importantly, hypothalamic metaflammation and microglial activation have been associated with hypothalamic resistance to peripheral anorexic signals, including leptin, and inhibition of hypothalamic inflammatory pathways reduces food intake and body weight of DIO animals (Zhang et al. 2008; Milanski et al. 2009; Posey et al. 2009). In addition, recent evidence suggests that depletion or inhibition of hypothalamic microglia prevents HFD-induced metaflammation and protects against DIO (Valdearcos et al. 2014; Andre et al. 2017; Thaler et al. 2012b; Valdearcos et al. 2017). Diet-induced obese rodents tend to reduce their body weight when switched to a less palatable diet (Myers Jr. et al. 2010). We have shown here that following transition back to standard chow diet, DIO mice normalize their weight, at mass, and glucose tolerance comparable to CD-fed controls. However, it is apparent from our results, as well as work done by others, that despite such weight loss hypothalamic metaflammation is slow to resolve (Wang et al. 2012; Enriori et al. 2007). Thus, while hypothalamic metaflammation is insufficient to maintain the full obesity phenotype in DIO animals in the absence of the primary precipitant (palatable high-calorie chow), persistent hypothalamic metaflammation and its ensuing leptin resistance could explain the accelerated weight gain and exacerbated metabolic derangements seen after weight cycling in rodent models (Barbosa-da-Silva et al. 2012; Thaiss et al. 2016), while in human obesity such mechanisms may contribute to the difficulty that most individuals have in achieving sustained weight loss in an environment with easy access to calorically-dense highly palatable foods (Anastasiou et al. 2015).

In contrast to the lingering hypothalamic metaflammation following dietary weight loss, our results show that hypothalamic metaflammation and the associated microgliosis are quick to resolve following two different bariatric surgical procedures, RYGB and BD-IL, despite ongoing exposure to HFD. The rapidity of these changes when compared to the DIO-Rev model suggests that these bariatric surgeries may elicit anti-inflammatory mediators that attenuate hypothalamic metaflammation and inhibit microglial activation, allowing for a reset of defended body weight to a new and lower level. Further studies are needed to assess the role, if any, the immediate post-operative anorexia and acute weight loss plays in the resolution of hypothalamic inflammation. It may be that following diet-reversal such inflammation may resolve given the same amount of time at a normal weight. However, other studies have shown that exposure to high-fat diet, and not obesity itself, rapidly induces hypothalamic inflammation (Yi et al. 2012).

In the setting of HFD-feeding, one would anticipate expression of hypothalamic anorexigenic genes, such as *Pomc*, to be upregulated while expression of orexigenic genes, such as *Agrp*, to be downregulated, reducing appetite, enhancing metabolism, and attenuating weight gain. Previous reports have demonstrated, however, that the expression levels of these hypothalamic leptin-responsive genes often counter the maintenance of homeostasis in the setting of DIO and increased serum leptin (Guan et al. 1998; Bergen et al. 1999; Huang et al. 2003), suggesting a degree of leptin resistance in the hypothalamic neurocircuitry. Consistent with these reports, we have observed that DIO-SHAM mice exhibit elevated hypothalamic expression of *Agrp* with no change in the expression of *Pomc* compared to CD-fed controls. We found that DIO-Rev mice exhibit a similar pattern of hypothalamic gene expression, although this pattern might be expected from animals who have recently lost weight (Yu et al. 2009). However, it also suggests that these animals continue to defend a higher body weight and would rapidly regain weight if returned to a more obesogenic environment (Barbosa-da-Silva et al. 2012; Thaiss et al. 2016). In contrast, the normalization of *Agrp* gene expression following both bariatric surgeries implies a state of homeostasis and is consistent with a reduced level of homeostatically defended body weight despite continued exposure to the same HFD as DIO-SHAM mice and a similar weight loss as DIO-Rev mice. Interestingly, hypothalamic AGRP neurons appear to be particularly sensitive to changes in the inflammatory environment and suppression of inflammatory pathways specifically in this neuronal population protects against DIO (Zhang et al. 2008), suggesting a mechanistic link between improved inflammation and *Agrp* levels.

Our results, while observational, generate important hypotheses regarding the relationship between hypothalamic metaflammation and the efficacy of bariatric surgery to induce sustainable weight loss. Changes in the anatomy and delivery of nutrients to the distal intestine following RYGB increase secretion of a number of metabolically active hormones and metabolites, some of which have anti-inflammatory properties which may act on hypothalamic cell types via direct or indirect mechanisms (le Roux et al. 2006). Recently, serum bile acids have emerged as important regulators of energy balance and possible mediators of bariatric surgery’s metabolic benefits (Kuipers and Bloks 2014; Albaugh et al. 2017). A number of studies in both human and animal models have shown increased serum bile acid concentrations following RYGB (Ahmad et al. 2013; De Giorgi et al. 2015; Pournaras et al. 2012; Bhutta et al. 2015; Spinelli et al. 2016). The BD-IL model of bariatric surgery, developed to investigate the effects of increased serum bile acids independent of surgical rearrangement of nutrient flow, results in weight loss and a reversal of DIO metabolic dysregulation, similar to that of RYGB (Flynn et al. 2015). Pertinent to our observations here, bile acids have potent anti-inflammatory effects via the G protein-coupled bile acid receptor GPBAR1 (Kawamata et al. 2003; Perino et al. 2014; Pols et al. 2011; Wang et al. 2011). Further, *Gpbar1* is expressed by microglia and GPBAR1 agonists have been shown to reduce neuroinflammation in several neurodegenerative diseases including models of multiple sclerosis, hepatic encephalopathy, and stroke (Lewis et al. 2014; Mano et al. 2004; Parry et al. 2010; McMillin et al. 2015; Rodrigues et al. 2002). Thus, we propose that bile acids may similarly mediate the anti-inflammatory effects of bariatric surgery on metabolically-induced hypothalamic inflammation. Further studies will be needed to define the cellular and molecular mechanisms underlying these effects, but such studies are likely to reveal important insights into the nature of hypothalamic inflammation and uncover novel targets for the non-surgical treatment of obesity.

## Conclusion

In summary, chronic high-fat diet (HFD) results in upregulation of inflammatory markers and proliferation of microglia within the mediobasal hypothalamus. We have shown here that two different models of bariatric surgery have an ameliorating effect on HFD-induced hypothalamic metaflammation and microgliosis while normalizing hypothalamic *Agrp* expression and the anorexigenic response to exogenous leptin, despite ongoing exposure to HFD. In contrast, animals that lost weight by transitioning back to a low-fat diet, continued to show evidence of hypothalamic inflammation and leptin resistance. These results suggest that bariatric surgeries may elicit anti-inflammatory mediators that counter the inflammatory effects of HFD and may be one reason for the efficacy of bariatric surgery in long-term weight loss. A better understanding of the mechanisms behind these effects may provide insight into novel therapies for obesity.

## Abbreviations

Agrp: Agouti-related peptide
ARC: Arcuate nucleus
BD-IL: Biliary diversion to the ileum
Ccl2: Chemokine (C-C motif) ligand 2
CD: Control diet
CX3CR1: Chemokine (C-X3-C motif) receptor 1
DAPI: 4′,6-Diamidine-2′-phenylindole dihydrochloride
DIO: Diet-induced obesity
DMH: Dorsomedial nucleus
GFP: Green fluorescent protein
GPBAR1: G protein-coupled bile acid receptor 1
HFD: High-fat diet
Il-1β: Interleukin 1 beta
MBH: Mediobasal hypothalamus
NF-κB: Nuclear factor kappa B
NMR: Nuclear magnetic resonance
PBS: Phosphate-buffered saline
PFA: Paraformaldehyde
Pomc: Pro-opiomelanocortin
RYGB: Roux-en-Y gastric bypass
SEM: Standard error of the mean
Tnfα: Tumor necrosis factor alpha
VMH: Ventromedial nucleus
αMSH: Alpha-melanocyte-stimulating hormone
